# Supplementary material for: Triggerable tough hydrogels for gastric resident dosage forms
Source: Nat Commun. 2017 Jul 25;8:124. doi: 10.1038/s41467-017-00144-z (PMC5527117; doi:10.1038/s41467-017-00144-z)
Supplement: Supplementary file 1 — Supplementary Information [file 41467_2017_144_MOESM1_ESM.pdf]

SI GUIDE

File Name: Supplementary Information
Description: Supplementary Figures.

File Name: Peer Review File
Description:

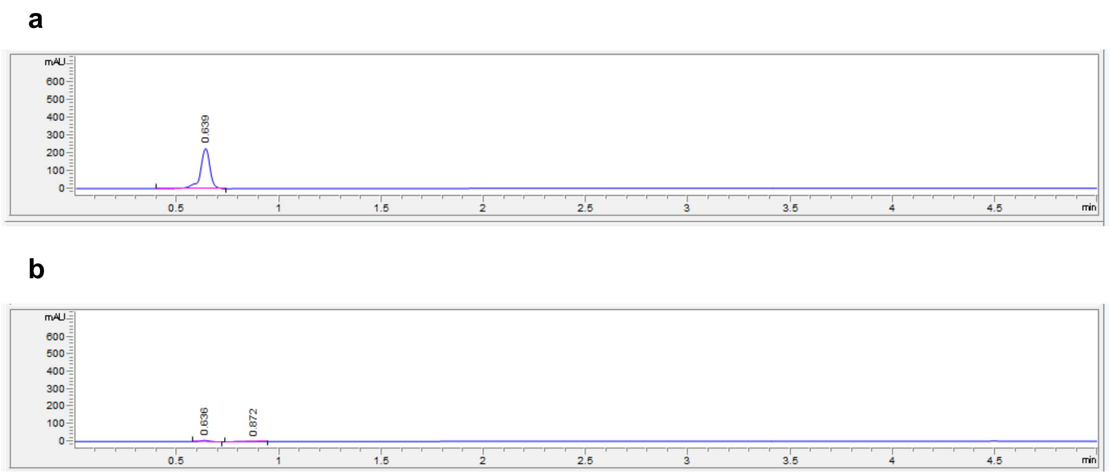

Supplementary Figure 1. Determination of the purity of TTHs. HPLC curves of the aqueous solutions extracted from the TTH (**a**) before and (**b**) after purification to show the complete removal of the unreacted acrylamide from the TTH.

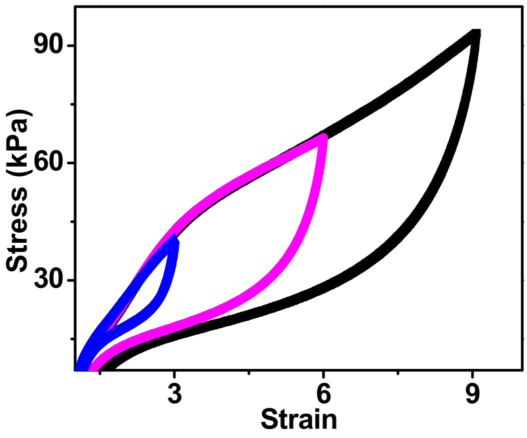

Supplementary Figure 2. Cyclic tensile test of TTHs. Samples of the TTH were subjected to a cycle of loading and unloading of varying maximum stretch.

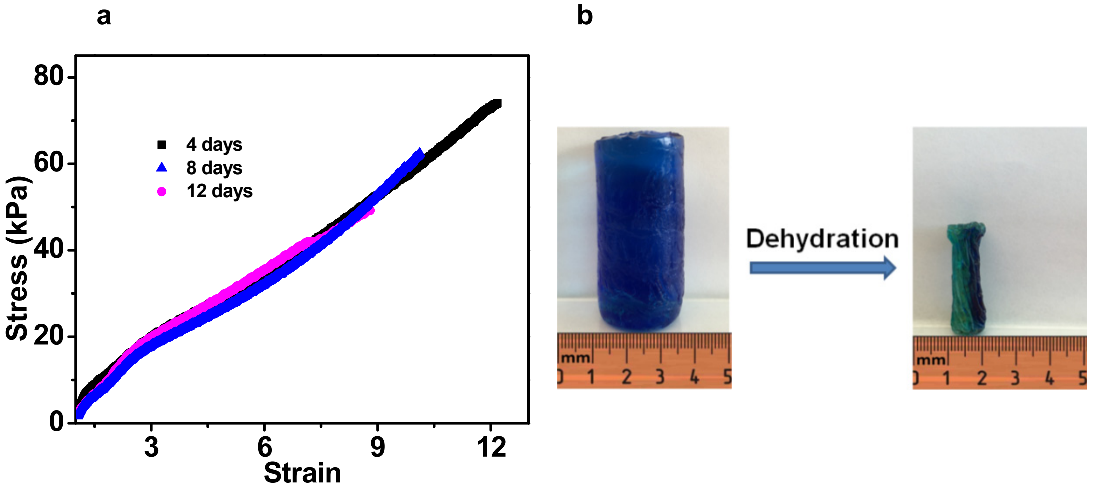

Supplementary Figure 3. Tensile test and dehydration of TTHs. (**a**) Tensile stress-strain curves of the TTH samples incubated in SGF at 37 °C for 4, 8, and 12 days. (**b**) Images of a cylindrical TTH sample dehydrated in air to 10 times its initial volume. The gel was labeled by methyl blue.

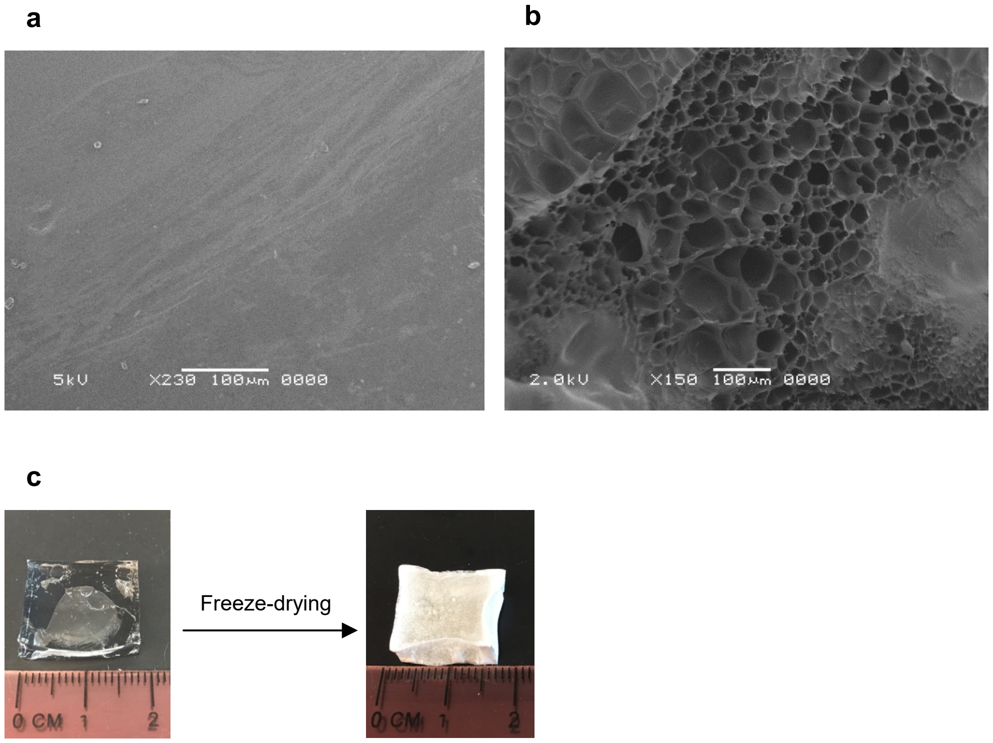

Supplementary Figure 4. SEM measurement and lyophilization of TTHs. Representative SEM images of the TTH samples dehydrated (**a**) in air and (**b**) by lyophilization. Scale bar: 100 µm. (**c**) Images of the TTH before and after freeze-drying.

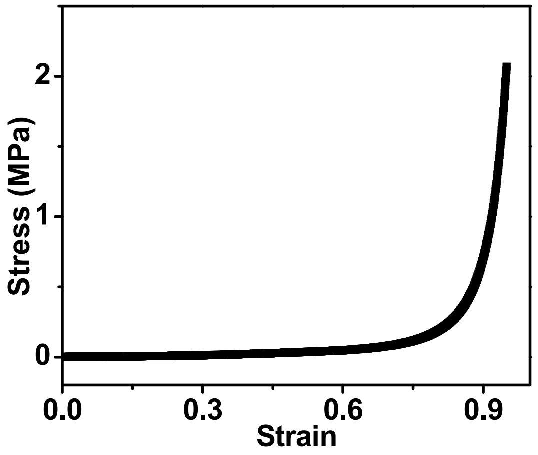

Supplementary Figure 5. Tensile test of TTHs. Compressive stress-strain curve of a TTH sample after a cycle of complete dehydration and subsequent rehydration.

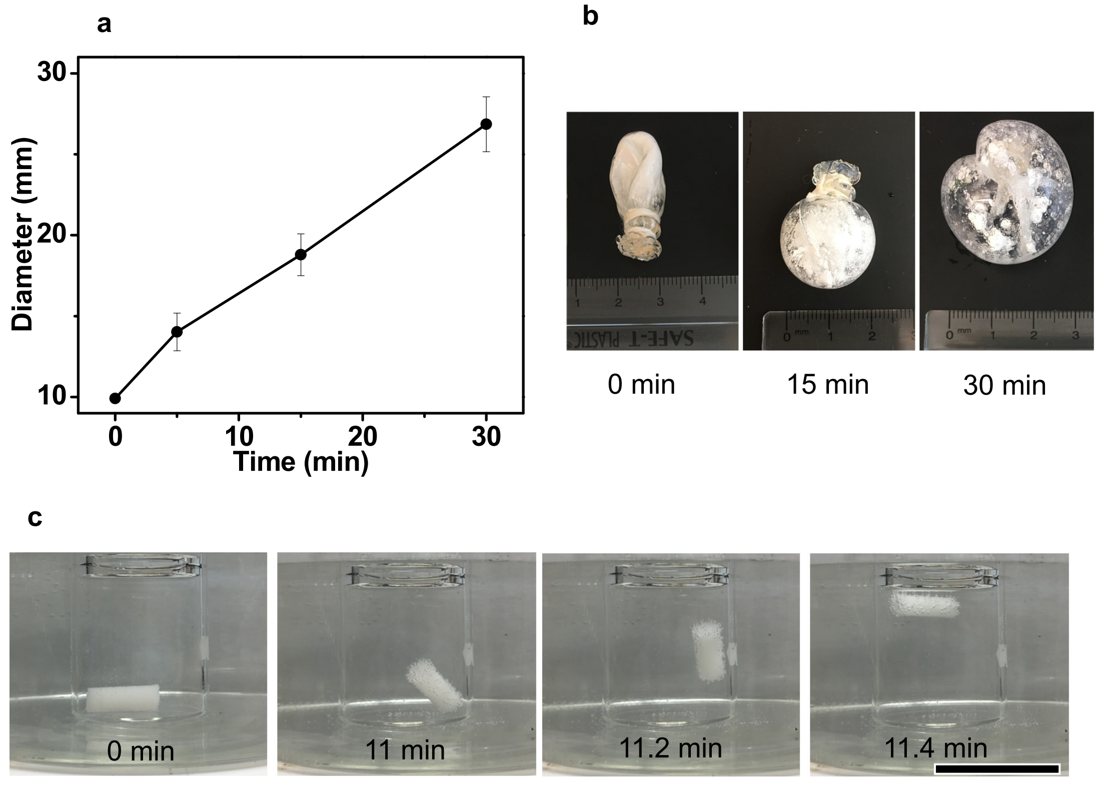

Supplementary Figure 6. Various dosage forms of TTHs. (**a**) Plot of diameter variation of the capsule-like TTH encapsulated with $CaCO_3$ inside (thickness of TTH: 1 mm) versus the incubation time at 37 °C in SGF. Error bars show standard deviation (n = 3). (**b**) Images of the capsule-like TTH encapsulated with $CaCO_3$ inside expanded in SGF at 37 °C. (**c**) A cylindrical TTH with 5 wt% $CaCO_3$ loading floated within 15 min in SGF at 37 °C. Scale bar: 2.5 cm.

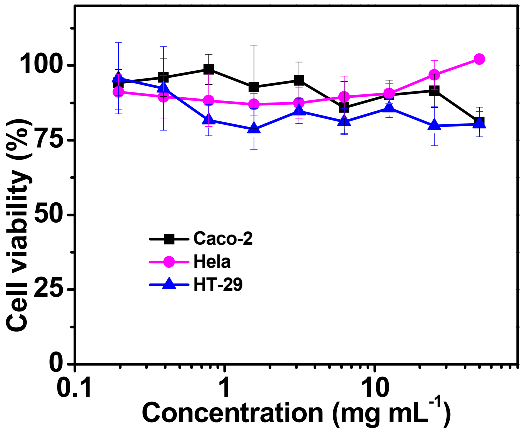

Supplementary Figure 7. Cytotoxicity of TTHs. Viability of cells cultured in the medium incubated with the TTH at 37 °C for 24 h with a dosage range from 0.2 to 50 mg mL^{-1}. The cells were incubated in the medium for 24 h. Error bars show standard deviation (n = 6).

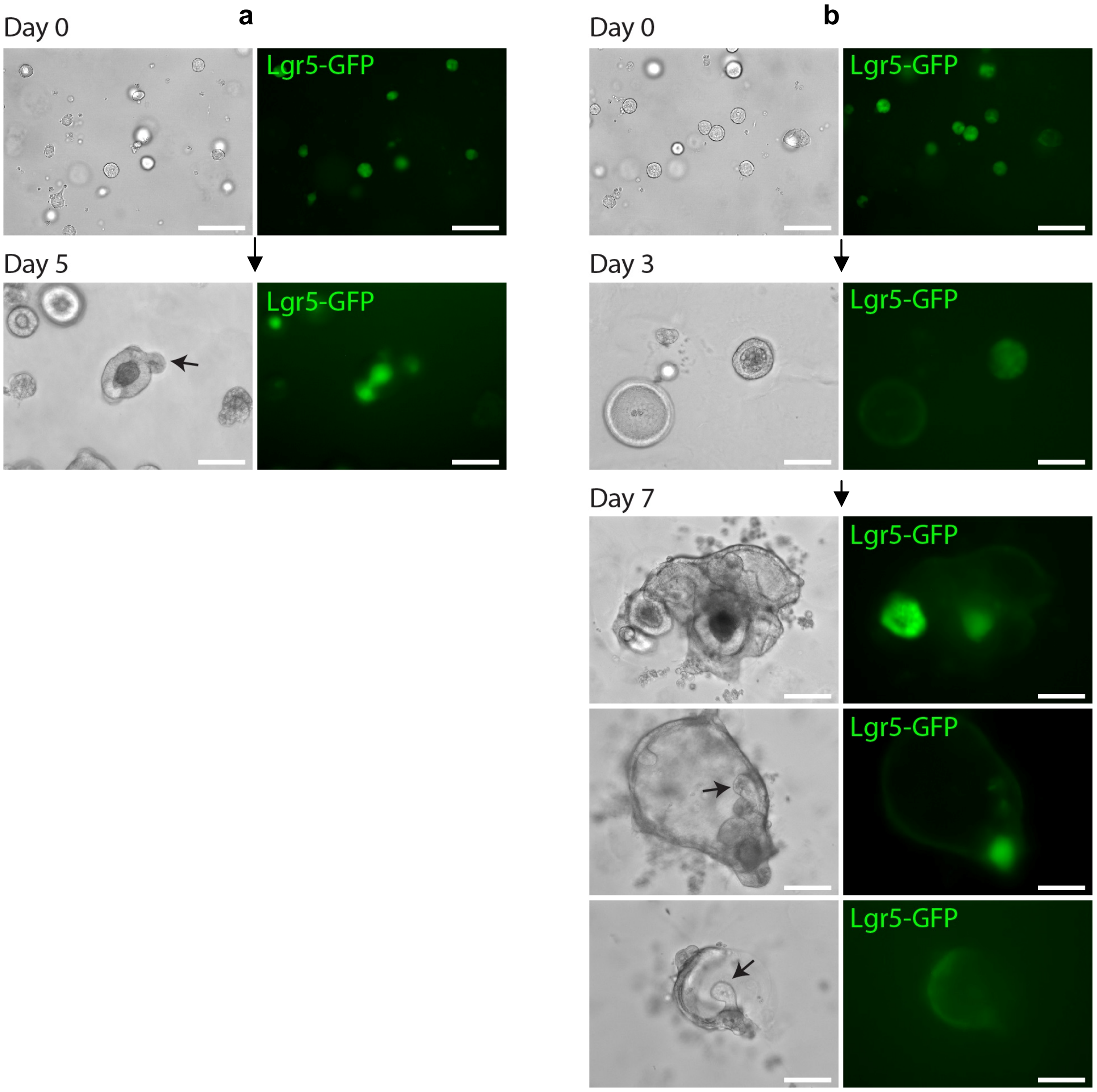

Supplementary Figure 8. Stem cell culture. (**a**) Co-culture of TTHs and mouse Lgr5+ intestinal stem cells showed low cytotoxicity of TTHs with stem cells over the course of 5 days. (**b**) Incubation of the $Lgr5^+$ stem cells on and within the TTHs indicated the cells retained their ability of multilineage differentiation to form organoids. Scale bar: 100 µm.

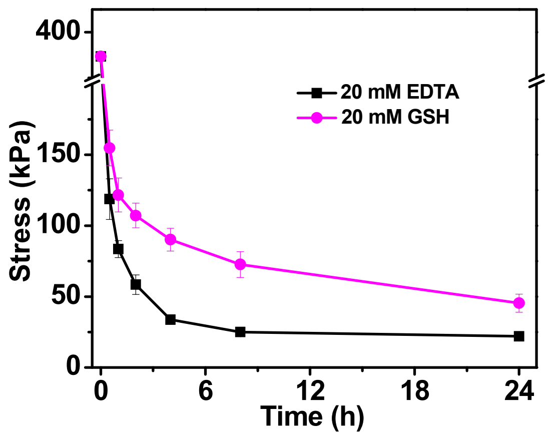

Supplementary Figure 9. Triggerable properties of TTHs. Plot of compressive stress of the TTH at strain of 80% versus the incubation time with 20 mM of EDTA or GSH at 37 °C. Error bars show standard deviation (n = 3).

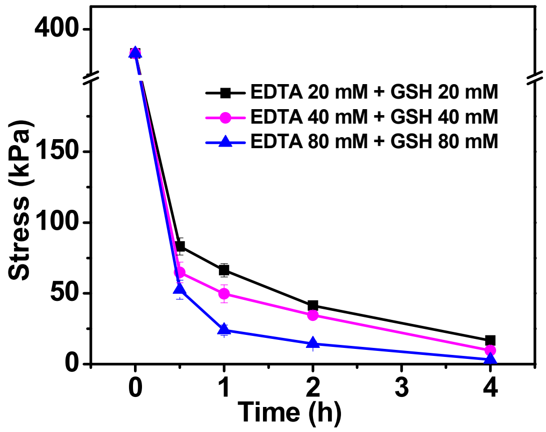

Supplementary Figure 10. Triggerable properties of TTHs. Plot of compressive stress of the TTH at strain of 80% versus the incubation time with EDTA and GSH in a range of concentration from 20 to 80 mM at 37 °C. Error bars show standard deviation (n = 3).

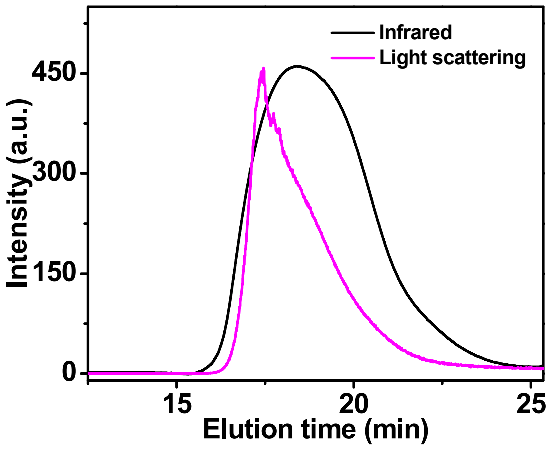

Supplementary Figure 11. GPC measurement of the dissolved TTH. GPC curves of the dissociated polymers from the TTH triggered by EDTA and GSH.

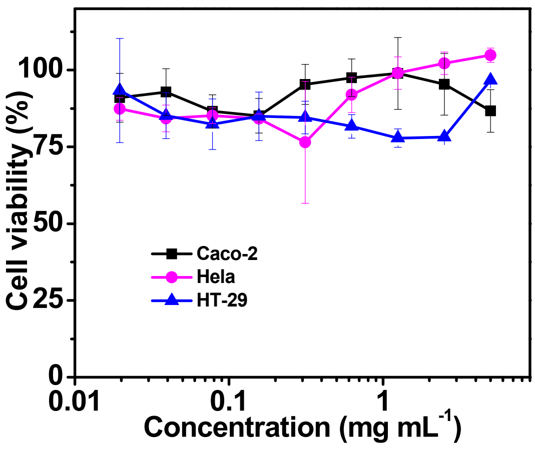

Supplementary Figure 12. Cytotoxicity of the dissociated TTH. Viability of cells cultured for 24 h in the medium with the dissociated TTH over a concentration range from 0.02 to 5 mg mL^{-1}. Error bars show standard deviation (n = 6).

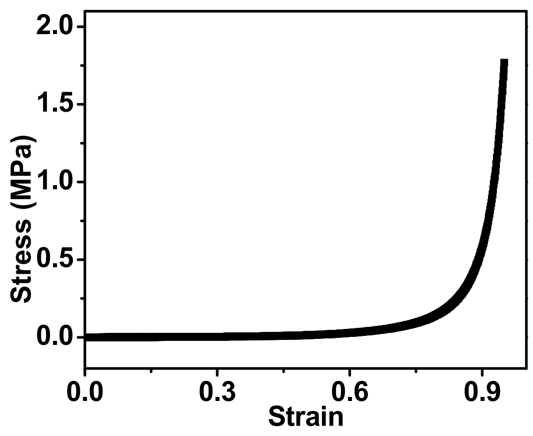

Supplementary Figure 13. Tensile test of the TTH strip. Compressive stress-strain curve of the TTH strip retrieved from the control pig.

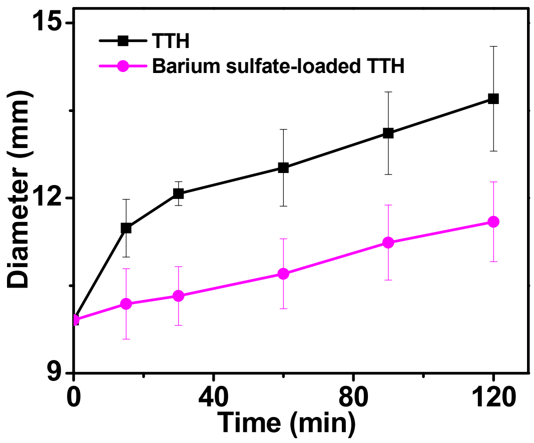

Supplementary Figure 14. Rehydration of the barium labeled TTH. Rehydration of dehydrated barium sulfide-loaded TTH in SGF at 37 °C. Dehydrated TTH was used as a control. Error bars show standard deviation ($n = 3$).

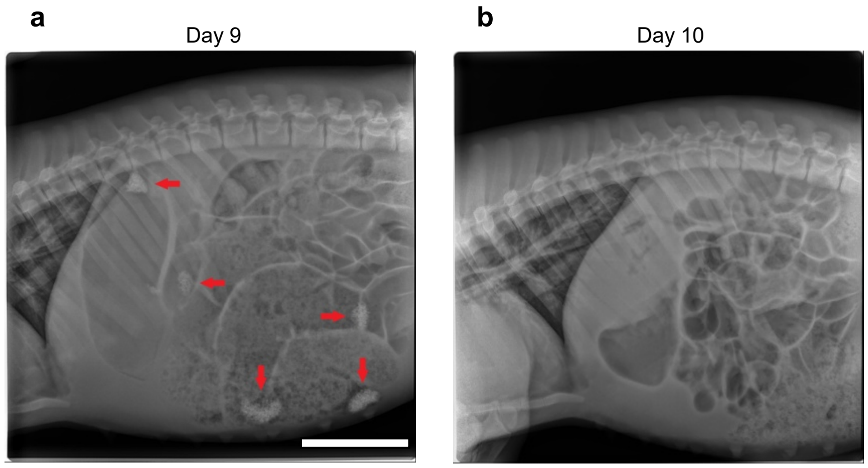

Supplementary Figure 15. Breakage of the TTH in stomach. Representative X-ray images of (**a**) the TTH device breakage in the gastric cavity and (**b**) the resulting fragments in the intestines of a Yorkshire pig as well as the safe pass of the fragments through the intestines in 24 h. Scale bar: 10 cm.

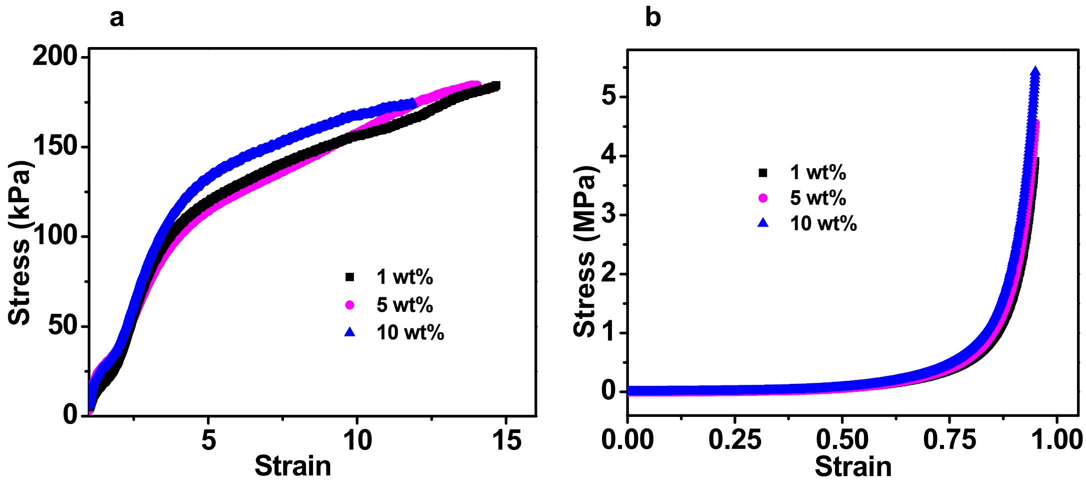

Supplementary Figure 16. Mechanical test of drug-loaded TTHs. (**a**) Tensile and (**b**) compressive stress-strain curves of the TTH loaded with various content of lumefantrine.

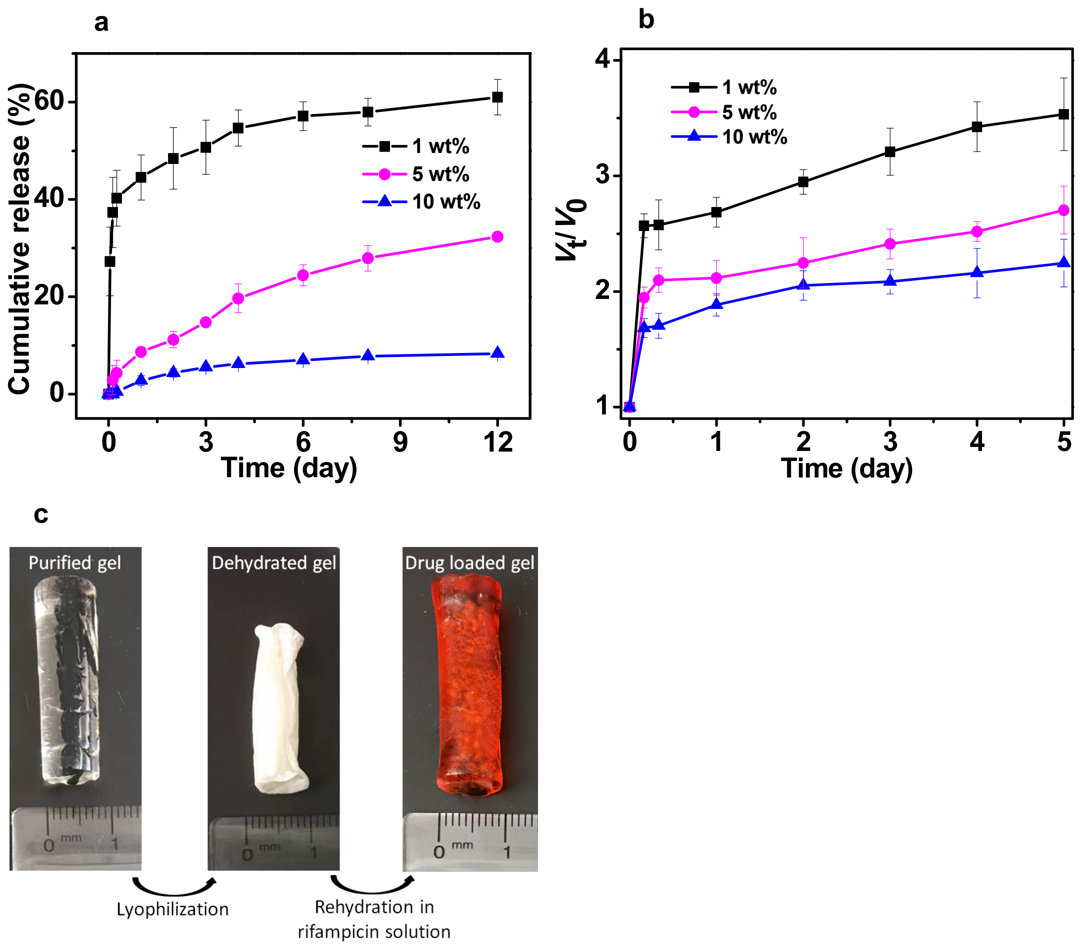

Supplementary Figure 17. Drug loading and release of TTHs. (**a**) The kinetics of release from the lumefantrine-loaded TTH in SGF at 37 °C. (**b**) The swelling kinetics of the drug-loaded TTH in SGF at 37 °C. Error bars show standard deviation (*n* = 3). (**c**) Preparation route of hydrophilic rifampicin-loaded TTH.

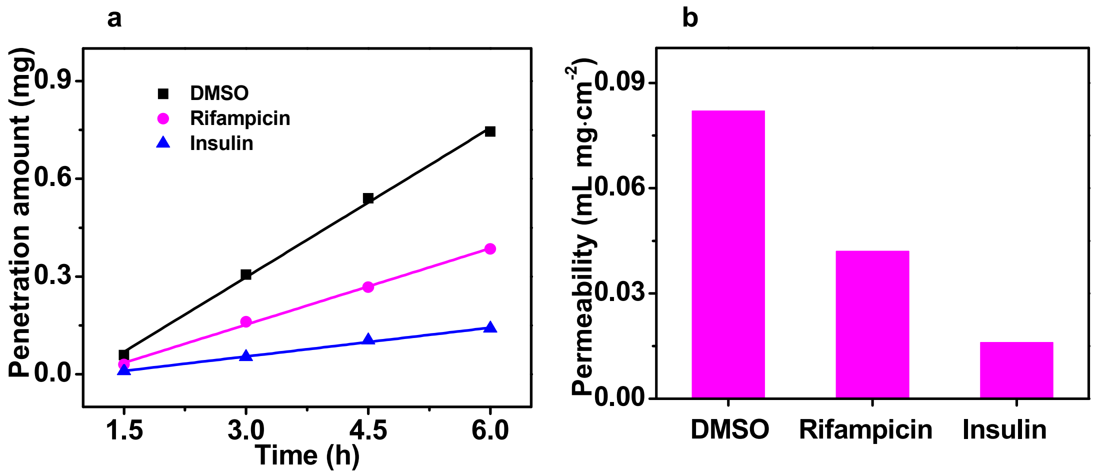

Supplementary Figure 18. Permeability measurement. (**a**) Plot of penetration amount through the TTH membrane (thickness: 3 mm) versus the incubation time. (**b**) The calculated permeability of DMSO, rifampicin, and insulin, respectively.

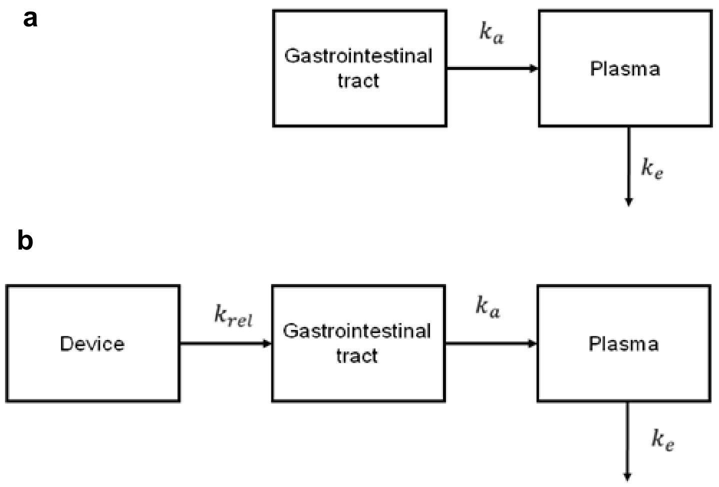

Supplementary Figure 19. Pharmacokinetic models. The model used to fit to the pharmacokinetic data of (**a**) free lumefantrine and (**b**) lumefantrine-loaded TTH device. The elimination rate constant and half-life were estimated to be 1.17 day^{-1} and 14.2 h for free lumefantrine as well as 0.68 day^{-1} (apparent elimination rate constant) and 24.3 h for lumefantrine-loaded device, respectively.
